# Adoptively transferred tumor-infiltrating T cells target somatic cancer mutations in a human papillomavirus+ cancer patient with complete tumor regression

**DOI:** 10.1186/2051-1426-3-S2-P52

**Published:** 2015-11-04

**Authors:** Sanja Stevanović, Pasetto Anna, Jared J Gartner, Eric Tran, Paul F Robbins, Steven A Rosenberg, Christian S Hinrichs

**Affiliations:** 1 Surgery Branch/National Cancer Institute / National Institutes of Health, Bethesda, MD, USA

## Background

Adoptive transfer of tumor-infiltrating lymphocytes (TIL) can mediate complete regression of metastatic cervical cancer, but the immunological landscape of the anti-tumor T cell responses in these patients is not fully understood. Reactivity against the human papillomavirus (HPV)-derived antigens may contribute to the clinical responses, but whether other immunogenic tumor antigens are also targeted by these effective TIL is unknown. Tumor-specific neo-antigens arising from somatically mutated genes are thought to be important for the therapeutic efficacy of immunotherapies in a variety of solid tumors. Cervical cancers also harbor somatic mutations that may be the targets of tumor-specific T cells.

Here we assessed whether mutated neo-antigens were the targets of clinically effective TIL in a patient with metastatic HPV16+ cervical cancer who experienced complete tumor regression, ongoing beyond 2 years, after TIL therapy.

## Methods

Whole-exome sequencing of a metastatic tumor was performed to identify tumor-specific non-synonymous somatic mutations. Subsequently, minigene constructs encoding 222 putative mutations (25-mer amino acid sequence with the mutated amino acid centrally located) were generated and transfected into autologous antigen presenting cells. Finally, reactivity of TIL against the transfected mutated minigene constructs was assessed by IFN-g ELISPOT, and up-regulation of T cell activation markers by flow cytometry.

## Results

The patient's infused TIL were previously reported to contain T cell reactivity against the HPV E6 and E7 antigens. Here, we found that these TIL also contained CD8+ T cell reactivity against three mutated neo-antigens encoded by the SETDB1, METTL17 and ALDH1A1 genes. The mutation-reactive CD8+ T cells showed exclusive recognition of their mutant 25-mer peptide over the wild-type counterpart, suggesting bona fide mutation-driven T cell reactivities. Furthermore, mutated neo-antigen T cell reactivities comprised ~24% of the infused TIL as assessed by CD137 up-regulation assay, and were mono- to oligoclonal as suggested by T cell receptor Vβ chain analysis of mutation-reactive bulk-isolated populations. Finally, no T cell reactivity against mutated antigens was detectable in peripheral blood pre-treatment, while mutation-reactive CD8+ T cells were present in peripheral blood at one month post-treatment. Analysis of mutation-derived neo-antigen T cell reactivities in additional HPV+ cancer patients treated with TIL therapy is currently ongoing.

## Conclusions

Our data reveal that mutated neo-antigens, in addition to previously reported HPV antigens, were the targets of clinically effective TIL in a patient with metastatic HPV+ cervical cancer who experienced complete tumor regression. This finding has important implications for future study and understanding of anti-tumor T cell responses in HPV+ cancers.

